# Endoscopic Diagnosis for *H. pylori* Infection: White Light Imaging (WLI) vs. Image-Enhanced Endoscopy (IEE)

**DOI:** 10.31557/APJCP.2021.22.9.3031

**Published:** 2021-09

**Authors:** Boonyaorn Chatrangsun, Ratha-Korn Vilaichone

**Affiliations:** 1 *Center of Excellence in Digestive Diseases and Gastroenterology Unit, Department of Medicine, Faculty of Medicine, Thammasat University Hospital, Pathumthani, Thailand. *; 2 *Department of Medicine, Chulabhorn International College of Medicine (CICM) at Thammasat University, Pathumthani, Thailand. *; 3 *Division of Gastroentero-Hepatology, Department of Internal Medicine, Faculty of Medicine, Universitas Airlangga, Surabaya, Indonesia. *

**Keywords:** Endoscopic diagnosis, white light imaging (WLI), image-enhanced endoscopy (IEE)

## Abstract

*Helicobacter pylori* infection is a class I carcinogen that can lead to gastric cancer. Early diagnosis and eradication of *H. pylori* infection are important to eliminate the risk of gastric cancer. Several invasive diagnostic techniques require biopsy samples, resulting in avoidable injury and medical expense. Furthermore, due to the localized distribution of *H. pylori*, random biopsies are not always reliable in diagnosing *H. pylori* infection. This article aimed to review endoscopic findings and new endoscopic options for the diagnosis of *H. pylori* infection. Using conventional white light imaging (WLI) and image-enhanced endoscopy (IEE), the endoscopic features associated with histological changes have increasingly become apparent. Real-time endoscopy is essential to make a diagnosis of *H. pylori* infection and allow targeted biopsy. Image-enhanced endoscopy (IEE), such as narrow-band imaging (NBI), linked color imaging (LCI), and blue laser imaging (BLI), enhances visualization of the surface vascular pattern and provides accurate diagnostic performance in *H. pylori* infection, as well as gastric neoplastic lesions, compared to conventional white light endoscopy. In conclusion, the new endoscopic technologies could be used in current practice with conventional white light endoscopy for accurate and real-time diagnosis of *H. pylori* infection and pre-cancerous lesions.

## Introduction

*Helicobacter pylori* infection is the leading cause of chronic gastritis, and it is classified as a class I carcinogen of gastric cancer by the World Health Organization (WHO) (Marshall, 2008). Early and accurate diagnosis of *H. pylori* infection is important for eliminating the risk of gastric cancer. Nowadays, there are invasive and non-invasive tests available to diagnose *H. pylori* infection. However, each technique has some limitations. For example, patients who use proton pump inhibitor PPI), antibiotics, anti-platelet, anti-coagulant, or direct oral anti-coagulant (DOAC) medications. Real-time endoscopy along with conventional white light imaging WLI) and image-enhanced endoscopic (IEE) techniques, such as narrow-band imaging (NBI), linked color imaging (LCI) and blue laser imaging (BLI), appear to have important roles in clinical practice to identify *H. pylori*-infected status (Malfertheiner et al., 2007). This article aimed to review the endoscopic diagnostic options and findings for *H. pylori* infection.

*Current diagnostic tests for H. pylori infection*

Many diagnostic tests are available, including invasive and non-invasive tests. Each method has advantages, disadvantages, and limitations of various clinical situations (Bray et al., 2018; Malfertheiner et al., 2007), which are demonstrated in Table 1. Real-time endoscopy has become an important tool for detecting *H. pylori* infection. It provides additional endoscopic information on gastric mucosal abnormalities and results in unnecessary mucosal injury and medical costs.

*Mechanism and equipment of endoscopic techniques for diagnosis of H. pylori infection*

Due to many limitations in the diagnostic tests for *H. pylori* infection, the development of new endoscopic techniques has provided reliable diagnostic tools for detection of *H. pylori* infection, pre-cancerous lesions, and gastric cancer.

*Conventional WLI*

The first case of flexible gastrointestinal (GI) endoscopy was performed in the 1960s (East et al., 2016), then advances in endoscopic technology have continued with high resolutions. Conventional white light endoscopy is the current standard for evaluating the mucosa of the GI tract due to accessibility, short endoscopic time, and low cost. In white light imaging, the normal gastric body is surrounded with folds, called rugae, which vary in size depending on the degree of insufflation. The mucosa of the fundus and antrum is normally smooth, and the color is velvety and red with regular arrangement of collecting venules (RAC). The RAC, mainly in the lesser curvature, were observed to be associated with *H. pylori*-negative gastric mucosa and a decreased risk of gastric cancer (Dohi et al., 2020). A magnifying (zoom) endoscopic technique shows normal fundic gland mucosa, including pit patterns and vascular details. There are consistent round or oval crypt openings in which pin-like dark spots are at the center of the gastric gland. The subepithelial capillary networks (SECNs) that surround the crypts have a honeycomb-like appearance (Sugano et al., 2015).

*NBI*

The first commercial narrow-spectrum technology, narrow-band imaging (NBI) (Olympus Medical Systems, Tokyo, Japan), was established in 2004. The narrow illumination is filtered by the function of NBI. The standard red, green, and blue (RGB) filters discard the red component, while the width of the spectral bands of the green and blue light is decreased from 50-70 nm to 20-30 nm. Narrow-band illumination is absorbed by hemoglobin, and the shortened wavelength penetrates the surface tissue. This technique results in enhanced contrast of superficial microvessels and mucosal surface (East et al., 2016). Magnifying narrow-band imaging (M-NBI) has widespread use in Asian countries but not in Western countries.

*LCI*

LCI (Lasereo; FUJIFILM Co., Tokyo, Japan) was launched in 2015. LCI is a color enhancement technology. The information on three colors (RBG) is used unlike the technique of WLI. The output of LCI provides the image with color enhancement in its range, enhancing the differences of mucosal color and helping to detect sufficient brightness (East et al., 2016). 

*BLI*

BLI (Lasereo; FUJIFILM Co., Tokyo, Japan) was first introduced in 2014. BLI functions with two types of lasers with wavelengths of 410 and 450 nm. The 450 nm laser conducts illumination light, which is similarly obtained with a xenon lamp. In BLI mode, the ratio of the BLI laser provides enhanced microvessels on the mucosal surface (Kato, 2016). Thus, its main role is observing the target at a short distance, which is called magnifying endoscopy. BLI-bright mode is a brighter BLI, consisting of BLI and white light mode laser illumination, and is mainly used for observing the target at middle and short distances. The high-intensity contrast imaging produced by magnifying blue laser imaging (M-BLI) provides clear visualization of microvascular and microsurface patterns like M-NBI.

*Histological findings and endoscopic findings of H. pylori infection*

*H. pylori* is a gram-negative microaerophilic spiral bacterium. *H. pylori* infection causes neutrophils and mononuclear cells to infiltrate the mucus neck region of the gastric mucosa and aggregate in the lumen of the pit. Chronic *H. pylori* gastritis results in continuous destruction and regeneration of pits and vessels. These ongoing processes can cause atrophic gastritis, intestinal metaplasia, dysplasia, and eventually gastric cancer (Wang et al., 2015).

According to the development of endoscopic technologies, many studies have demonstrated that endoscopic features were associated with histological findings (Toyoshima et al., 2020). Table 2 describes the findings (Kato, 2016). Endoscopic techniques, such as conventional white light imaging (WLI) and image-enhanced endoscopy (IEE), have become reliable diagnostic modalities for *H. pylori* infection.

*Diagnostic performance of H. pylori infection in various endoscopic techniques and clinical applicability*

Each endoscopic technique could be used for identifying *H. pylori* infection by using the specific endoscopic features. Meanwhile, some endoscopic features could be used for excluding *H. pylori* infection. A summary of the endoscopic features of *H. pylori*-positive and *H. pylori*-negative gastric mucosa in various techniques are demonstrated in Table 3 and Table 4, respectively.

*Conventional WLI*

**Figure 1:**
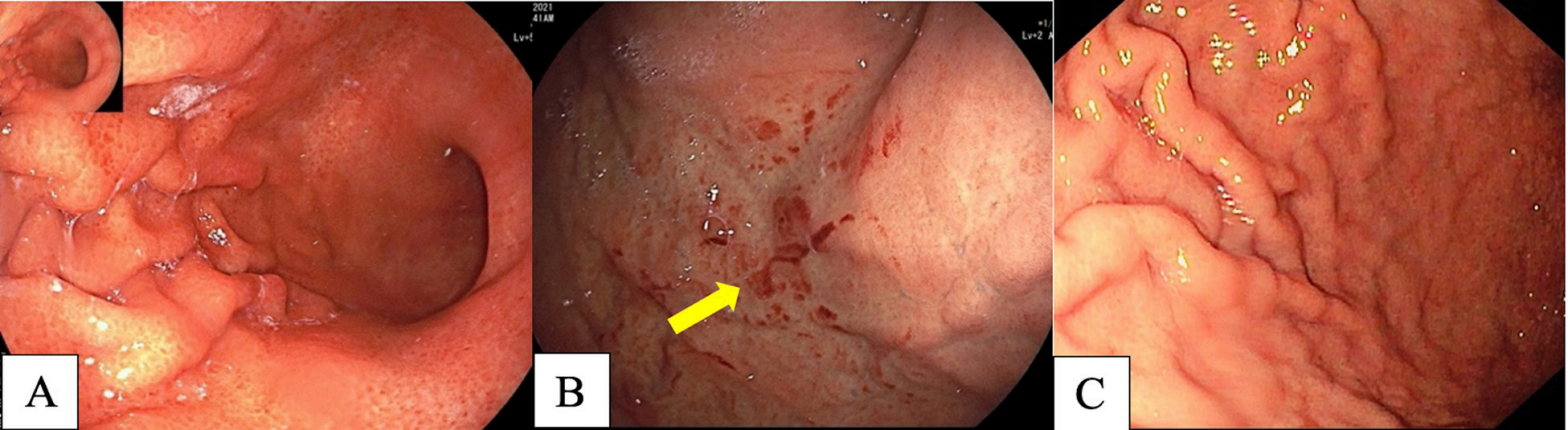
*H. pylori*-Positive Gastritis in Conventional White Light Imaging (WLI). A, diffuse redness of gastric mucosa; B, spotty hemorrhage at fundus (arrow); C, enlarged gastric folds

**Figure 2:**
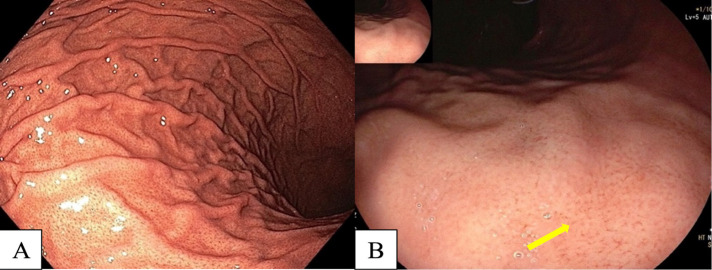
A, Regular Arrangement of Collecting Venules (RAC) at Body; B, Near Focus of RAC (arrow).

**Figure 3:**
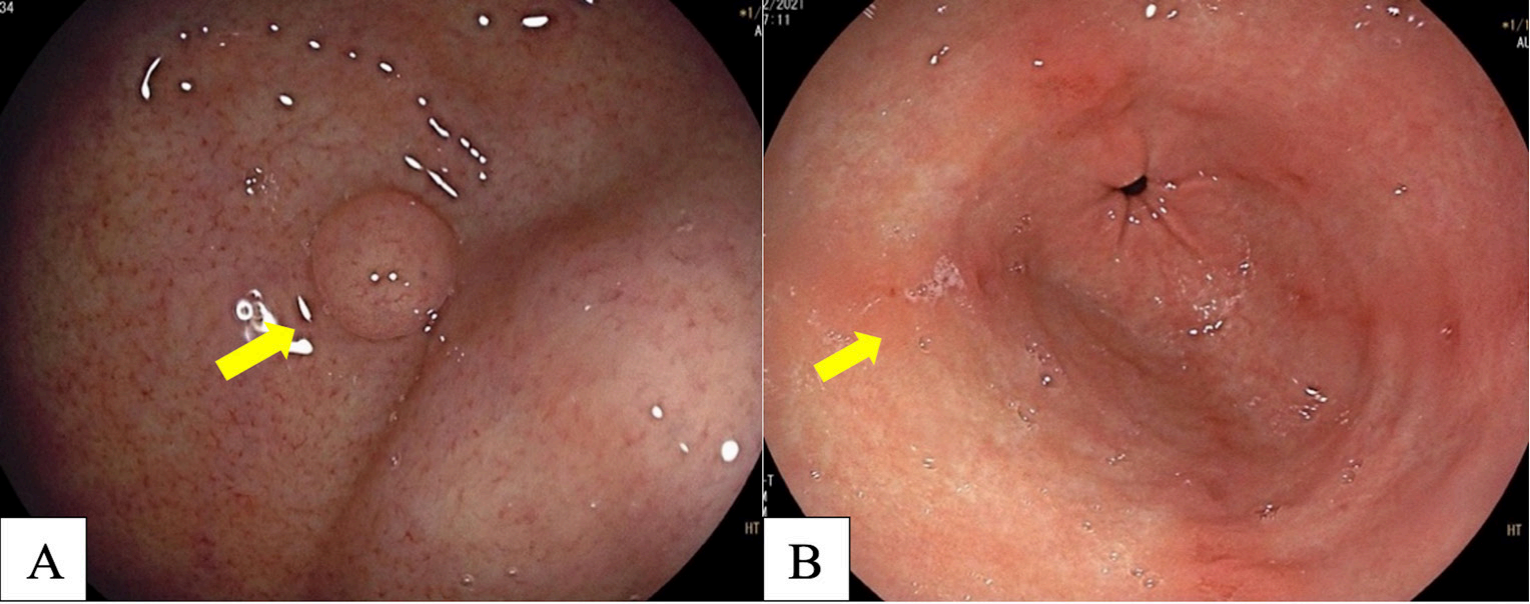
A, Fundic Gland Polyp (arrow); B, Red Streak (arrow).

Many studies have reported diffuse redness of the gastric mucosa, spotty hemorrhage at the fundus, enlarged gastric folds, and sticky mucus and antral nodularity in conventional white light imaging (WLI) were associated with *H. pylori*-positive gastric mucosa with a sensitivity/specificity of 57.52%/95.8%, 61.06%/95.8%, 60.18%/92.25%, 53.33%/95.1%, and 100%/100%, respectively (Nishizawa et al., 2020; Ono et al., 2020). Figure 1 demonstrates patterns of *H. pylori*-positive gastric mucosae. On the other hand, the presence of RAC in the corpus10 (Figure 2), fundic gland polyps, and red streaks (Figure 3) were associated with *H. pylori*-negative gastric mucosa with a sensitivity/specificity of 92.4%/94.5%, 20.4%/96.9%, and 19.5%/95.4%, respectively (Zhao et al., 2020).

According to the inconsistent results of many studies, the Kyoto consensus meeting focused on endoscopic findings to accurately determine *H. pylori* infection using summation of the scores for endoscopic findings, such as gastric atrophy, intestinal metaplasia, enlarged gastric folds, antral nodularity, and RAC. 

The Kyoto classification has been defined as follows: a score of 0 indicates *H. pylori*-negative gastritis and a score ≥2 indicates *H. pylori*-positive gastritis, with an accuracy of 90% (Toyoshima et al., 2020).

*IEE*

Advanced endoscopic imaging can improve mucosal and vascular visualization, especially in magnifying mode. Magnifying endoscopy, especially NBI and BLI, could enhance fine structural and microvascular detail (pit plus vascular pattern) (Qi et al., 2016). Many clinical studies have reported IEE could help identification of mucosal changes and be used for precise targeted biopsies. Limitations of using IEE include needing more training and learning curve for experiences, as well as it being time consuming.

*LCI*

**Figure 4:**
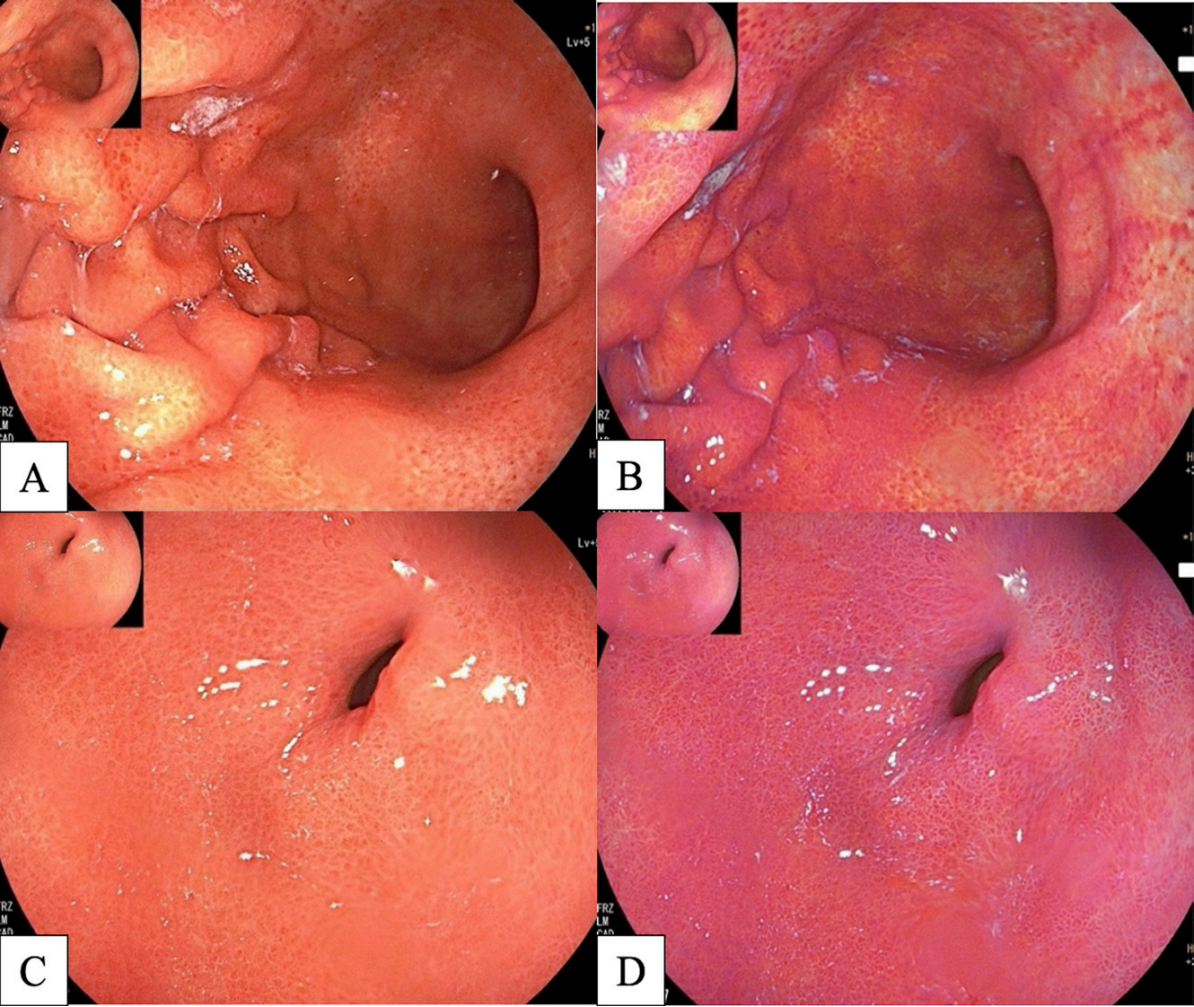
A, Diffuse Redness in Gastric Body in WLI; B, Diffuse Redness in Gastric Body (Deep Reddish Color) in LCI; C, Diffuse Redness in Gastric Antrum in WLI; D, Diffuse Redness in Gastric Antrum (Deep Red Color) in LCI

**Figure 5:**
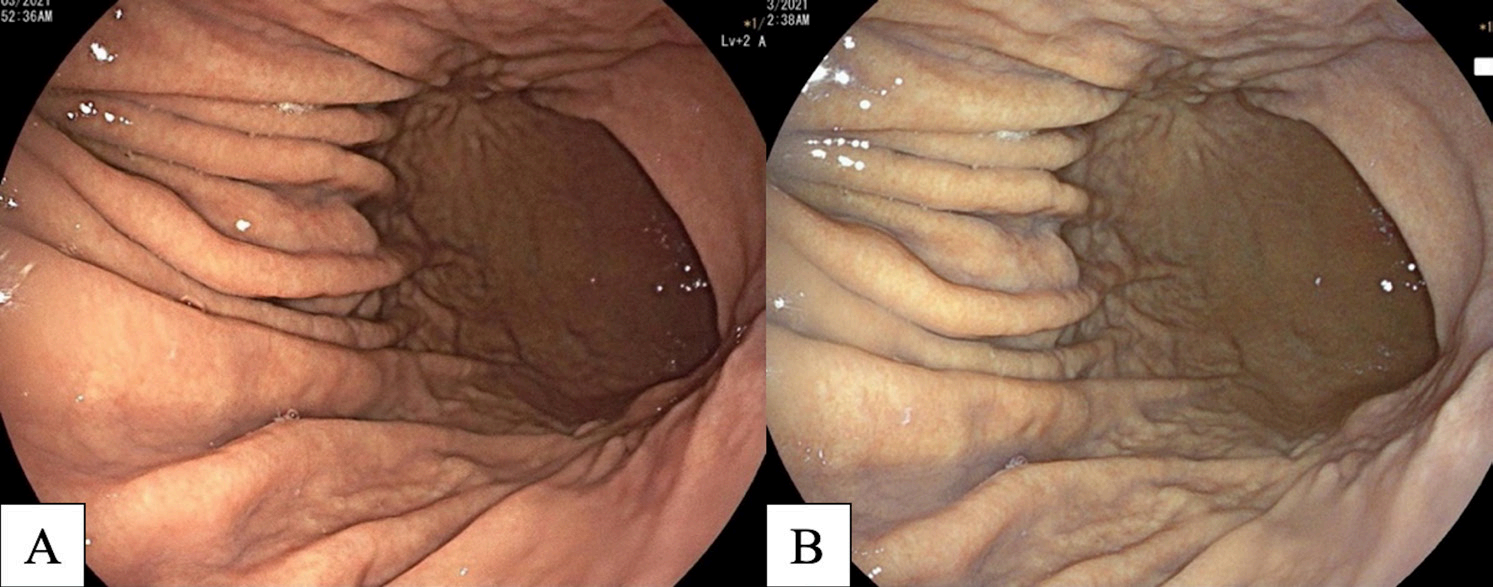
A, *H. pylori*-Negative Infection Gastric Mucosa in WLI; B, Light Orange/White Apricot Gastric Mucosa in LCI

LCI (FUJIFILM Co., Tokyo, Japan) is an IEE technique using a laser light source. LCI provides an approximate color difference twice as high as in WLI. Recent studies have reported LCI produces three times greater amplification to distinguish abnormal lesions from normal mucosa. As is demonstrated in Figure 4, WLI shows diffuse redness over the entirety of the gastric mucosa, while LCI shows deep reddish mucosa over the entirety of the stomach, implying *H. pylori*-positive mucosa. In the case of *H. pylori*-negative mucosa, WLI shows yellowish mucosa over the entirety of the gastric mucosa, while LCI shows a light orange (white apricot) hue over the entirety of the gastric mucosa, as is demonstrated in Figure 5. Retrospective studies in Japan (Yagi, Aruga, Nakamura, & Sekine, 2005) showed LCI was more accurate at identifying *H. pylori*-positive mucosa than WLI, with a sensitivity of 93.3.8% and a specificity of 78.3% (Dohi et al., 2016; Sun et al., 2016). 

*NBI*

Generally, NBI is used in combination with magnifying mode (M-NBI). A normal gastric corpus mucosal surface is composed of round or oval crypt openings. Dark brownish spots in the crypt openings are at the center of the gastric gland. The subepithelial collecting networks (SECNs) surrounding the crypts have a honeycomb-like appearance with RAC.

**Figure 6:**
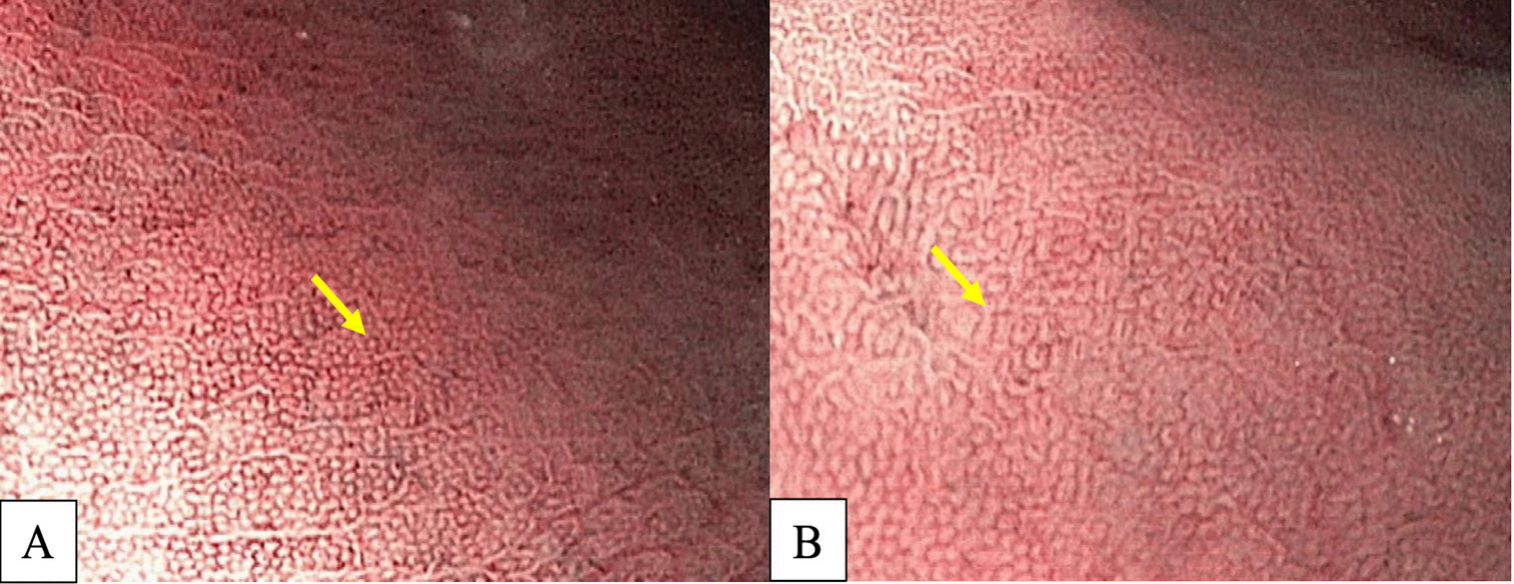
A,* H. pylori*-Negative Gastric Mucosa is Characterized by Homogeneous, Round Pits with Regular Honeycomb-Like SECNs in NBI; B, *H. pylori-*Positive Gastric Mucosa is Characterized by Enlarged or Elongated, Varies in Sized and Shaped of Pits with Unclear SECNs in NBI

In *H. pylori*-related gastritis, the edematous mucosa results from infiltration of neutrophils and mononuclear cells. Pits are enlarged or elongated due to destruction of the vessels and increased density of irregular microvessels (Horiguchi et al., 2017). The collecting venules are obliterated due to inflammation. The sensitivity and specificity of magnifying NBI (M-NBI) endoscopy for detecting *H. pylori* infection is high with 97% and 81%, respectively (Tahara et al., 2019; Tahara et al., 2009). Figure 6 demonstrates *H. pylori*-negative and *H. pylori*-positive gastric mucosae. 

*BLI*

**Figure 7:**
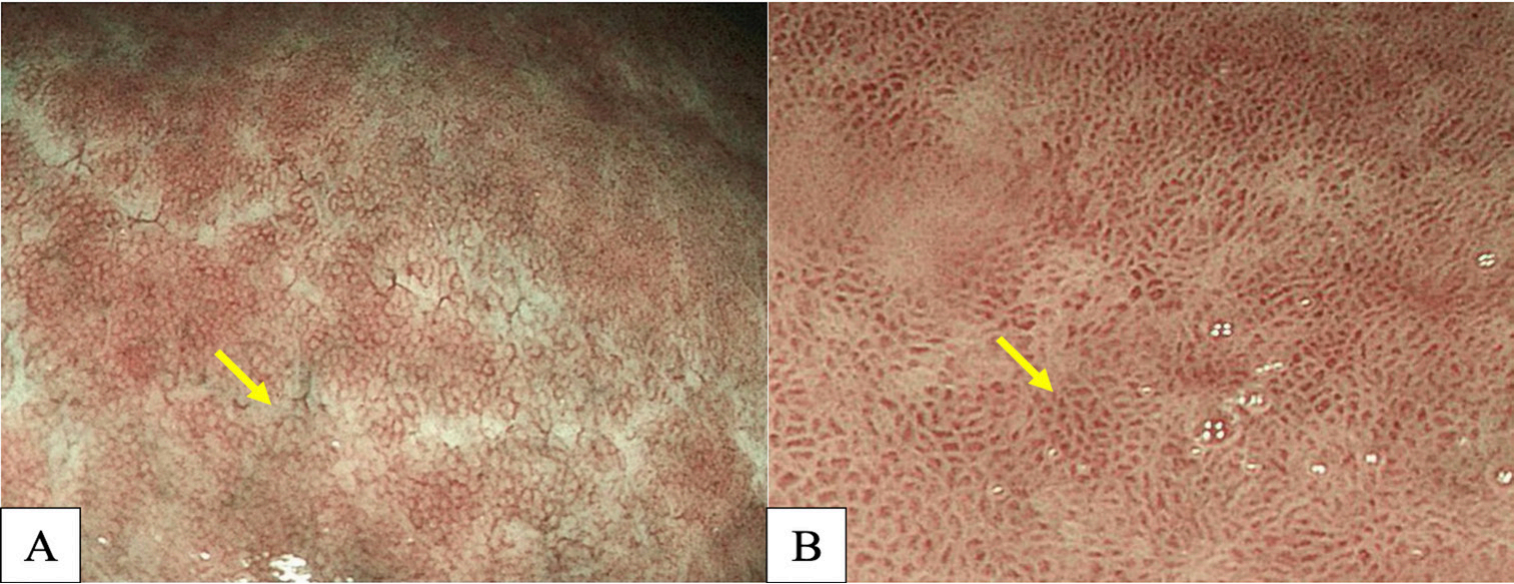
A, *H. pylori-*Negative Gastric Mucosa is Characterized by Homogeneous, Round Pits with Regular Honeycomb-Like SECNs in BLI; B, *H. pylori*-Positive Gastric Mucosa is Characterized by Enlarged or Elongated, Varied in Sized and Shaped of Pits with Unclear SECNs in BLI

The NBI technologies can be limited by a dark field of view. However, the BLI system provides laser light system shows a clearer view with high contrast of the gastric mucosa and vascular structures. Similarly, in NBI, M-BLI patterns of gastric mucosa are associated with histological findings of *H. pylori* infection. Moreover, it is also useful for distinguishing *H. pylori*-related gastritis, as is magnifying NBI (M-NBI). M-BLI endoscopy has potential diagnostic performance for *H. pylori*-related gastritis with a sensitivity of 98% and a specificity of 92% (Tomomitsu Tahara et al., 2017), as is demonstrated in Figure 7.

*Clinical study in Thailand*

Our randomized prospective study conducted at Thammasat University Hospital, Thailand, during 2020-2021, is the first study comparing each endoscopic technique, simultaneous EGD using WLI, LCI, NBI and BLI, for the diagnosis of *H. pylori* infection. We found that the endoscopic features associated with *H. pylori* infection were diffuse redness, enlarged gastric folds and sticky mucus (positive predictive value [PPV]: 83.3%, 100% and 100%, respectively). RAC had a high negative predictive value (NPV) (88%) for excluding *H. pylori* infection. The sensitivity, specificity, PPV, NPV and accuracy for diagnosis of *H. pylori* infection using WLI, LCI, NBI, and BLI are demonstrated in Table 5. Moreover, additional IEE to conventional WLI could improve the diagnostic performance of *H. pylori* infection in our study.

*New innovative tool (EndoFaster)*

EndoFaster (NISO Biomed S.r.l., Turin, Italy) was first introduced in 2005. EndoFaster is a real-time analysis machine using gastric juice that provides information on ammonium concentration and gastric pH (Sánchez Rodríguez et al., 2020). Because *H. pylori* can produce the urease enzyme, which breaks down urea into carbon dioxide and ammonia, this machine could diagnose *H. pylori* infection through a urease test on gastric juice. A total of 2-4 ml of gastric juice was aspirated during EGD and analyzed by the EndoFaster within 1 minute (Costamagna et al., 2016). Many studies about using the EndoFaster for the real-time diagnosis of *H. pylori* infection have reported a high accuracy, which is comparable to the urea breath test (UBT). One large prospective study conducted in Italy, which compared the EndoFaster and urea breath test (UBT) with histological examination as the gold standard for diagnosis of *H. pylori* infection, demonstrated a sensitivity of 90.3% and a specificity of 85.5%. Moreover, the overall benefits of this device include being less invasive, not requiring proton pump inhibitor (PPI) discontinuation before testing, and less costs. Recent studies have demonstrated the EndoFaster has advantages in detection of hypochlorhydric conditions, neoplastic risk conditions, and as an adjunct to gastroesophageal reflux (GERD) treatment (Zullo et al., 2021).

*Artificial intelligence for predicting H. pylori infection in endoscopic images*

Artificial intelligence (AI) has been recently introduced and increasingly used in clinical practice. The diagnostic performance of AI is used in endoscopic images to detect pre-cancerous and cancer lesions. The application of AI in *H. pylori* infection is to decrease interobserver disagreement and time consumption (Pannala et al., 2020). The development of AI potentially detects *H. pylori* infection by integrating data into endoscopic images. The innovation of AI is to mimic human neural networks in the brain. AI could analyze images for many features, including sizes, shapes, colors, and even textures. Most of the studies on the application of AI in endoscopic practices are in Japan because of the high incidence of *H. pylori* infection and burden of gastric cancer screening (Bang et al.,, 2020). 

One large prospective randomized controlled study in Japan compared accuracy in the diagnosis of *H. pylori* infection between experienced endoscopists and AI. A total of 32,208 endoscopic images in eight important areas of stomach were categorized as having *H. pylori*-positive or *H. pylori*-negative status. The results of this study found that AI has greater sensitivity in the diagnosis of *H. pylori* infection than experienced endoscopists, with a sensitivity and specificity of 81.9%/83.4% and 79%/83.2%, respectively (Nakashima et al., 2018). On the other hand, studies of AI in IEE have been increasing. Another prospective pilot study conducted in Japan, compared AI-assisted BLI-bright, LCI and WLI in the diagnosis *H. pylori* infection. The area under the curve (AUC) of AI-BLI-bright and AI-LCI were 0.96 and 0.95, respectively, whereas AI-WLI had and AUC of 0.66 (Nakashima et al., 2018). Nowadays, AI technology has become a useful diagnostic tool for endoscopists, especially when using it with IEE. AI provides a second opinion, some important findings during endoscopy, decreased time consumption, and less of a learning-experience requirement. AI might be an excellent future diagnostic modality for the diagnosis of *H. pylori* infection.

In conclusion, developments of endoscopic techniques contribute to the real-time diagnosis of *H. pylori* infection during endoscopy. Endoscopic imaging can reflect histological features of the gastric mucosa. WLI seems to be a good modality for the diagnosis of *H. pylori* infection because of its widespread use, short endoscopic time, and requirements of less experience (Glover et al., 2020). Endoscopic findings, including diffused redness of gastric mucosa, and spotty hemorrhage at fundus, were strongly suggestive of *H. pylori*-positive status. On the other hand, RAC is associated with an absence of *H. pylori* infections by WLI with high sensitivity and specificity. Using IEE can improve mucosal, fine structural, and microvascular visualization, especially use with M-BLI endoscopy, which could provide a high potential diagnostic performance for *H. pylori*-related gastritis. The aforementioned techniques could accurately diagnose *H. pylori* infection and pre-cancerous lesions (targeted biopsy) better than the current practice with conventional WLI.

**Table 1:** Sensitivity, Specificity, Advantages, and Disadvantages of Diagnostic Tests for Detecting *H. pylori* Infection

Diagnostic test	Sensitivity	Specificity	Advantages	Disadvantages
Rapid urease test (RUT)	93 - 97 %	98%	- Fast	- Invasive - False negative in ATB, PPI usage, and GI bleed
Histochemical staining test	80 - 90 %	90 - 100 %	- Gold-standard	- Invasive and need pathologist
Urea breath test	90 - 97 %	95 - 100 %	- Non-invasive - Confirm eradication of treatment	- Expensive - False negative in ATB, PPI usage, and GI bleed
Stool antigen test	92.20%	94.40%	- Inexpensive - Confirm eradication of treatment	- False positive in PPI usage - Difficult to carry specimen
*H. pylori *antibody with current infection (CIM)	90 - 95%	90 - 95 %	- Fast	- Not in widespread use
*H. pylori *culture	85 - 95%	99 - 100%	- Provide ATB resistance	- Expensive, need expertise, not widespread use
PCR for *H. pylori*	95%	95 - 100%	- Fast	- Expensive, false positive result - Risk for contamination

ATB, antibiotics; GI, gastrointestinal; *H. pylori*, Helicobacter pylori; PPI, proton pump inhibitor; PCR, polymerase chain reaction

**Table 2:** Relationship between Histological and Endoscopic Findings of *H. pylori* Infection (Suzuki et al., 2016).

Histological findings	Endoscopic findings
Mucosal hyperemia	Erythema
Mucosal edema	Mucosal swelling
Mucosal epithelial defect	Erosions and ulcers
Mucosal hemorrhage	Bleeding spot
Infiltration of polymorphonuclear cells and mononuclear cells	Diffuse redness and disappearance of RAC
	Visibility of vascular pattern and rugal atrophy
Mucosal atrophy	Whitish elevated lesion (specific type)
Intestinal metaplasia	Light blue crest (by IEE)
	Marginal turbid band
	White opaque substance (by IEE)

RAC, regular arrangement of collecting venules; IEE, image-enhanced endoscopy

**Table 3:** Summary of Endoscopic Features of *H. pylori*-Positive Gastric Mucosa in Various Techniques (Chatrangsun et al., 2021; Mao et al., 2016; Tomomitsu Tahara et al., 2017).

Endoscopic technique	*H. pylori*-positive gastric mucosa		
	Endoscopic features	Sensitivity	Specificity
WLI	Diffuse redness	57.50%	95.80%
	Antral nodularity	100%	100%
	Spotty hemorrhage at fundus	61.00%	95.80%
	Enlarged gastric folds	60.10%	92.20%
	Sticky tenacious mucus	53.30%	95.10%
	Xanthoma	11.20%	98.00%
LCI	Diffuse redness (deep red color)	93.30%	78.30%
	Antral nodularity	25%	100%
	Spotty hemorrhage at fundus	50%	100%
	Enlarged gastric folds	15%	100%
	Sticky tenacious mucus	5%	100%
	Xanthoma	5%	100%
NBI	Elongated pits, variable sizes and shapes	N/A	N/A
	Obliterated collecting venules	97.00%	81.00%
BLI	Elongated pits, variable sizes and shapes	N/A	N/A
	Obliterated collecting venules	98.00%	92.00%

BLI, blue laser imaging; *H. pylori*, Helicobacter pylori; LCI, linked color imaging; NBI, narrow-band imaging; WLI, white light imaging

**Table 4:** Summary of Endoscopic Features of *H. pylori*-Negative Gastric Mucosa in Various Techniques

Endoscopic technique	*H. pylori*-negative gastric mucosa		
	Endoscopic features	Sensitivity	Specificity
WLI	RAC	92.40%	94.50%
	Fundic gland polyp	14.60%	95.50%
	Hematin spots	12.80%	93.80%
	Red streaks	100%	2.80%
	Raised erosion	2.80%	99.10%
LCI	Light orange/white apricot mucosa	96.70%	50%
	RAC	76.70%	90%
	Fundic gland polyp	13.30%	100%
	Hematin spots	16.70%	100%
	Red streaks	16.70%	100%
	Raised erosion	10%	100%
NBI	Round homogenous sized pits and presence of RAC	80%	85%
BLI	Round homogenous sized pits and presence of RAC	80%	95%

BLI, blue laser imaging; *H. pylori,* Helicobacter pylori; LCI, linked color imaging; N/A, not available; NBI, narrow-band imaging; RAC, regular arrangement of collecting venules; WLI, white light imaging.

**Table 5:** Sensitivity, Specificity, PPV, NPV, and Accuracy of Each Endoscopic Technique for Diagnosis of *H. pylori* Infection (Chatrangsun et al., 2021)

Endoscopic technique	Sensitivity	Specificity	PPV	NPV	Accuracy
	(95% CI)	(95% CI)	(95% CI)	(95% CI)	(95% CI)
WLI	90.00% (68.3-98.8)	70.00% (50.6-85.3)	66.70% (53.2-77.9)	91.30% (73.4-97.6)	78.00% (64.0-88.5)
LCI	95.00% (75.1-99.9)	76.70% (57.7-90.1)	73.10% (58.5-84.0)	95.80% (77.1-99.4)	84.00% (70.9-92.8)
BLI	95.00% (75.1-99.9)	80.00% (61.4-92.3)	76.00% (60.6-86.7)	96.00% (77.9-99.4)	86.00% (73.3-94.2)
NBI	85.00% (62.1-96.8)	80.00% (61.4-92.3)	73.90% (57.5-85.6)	88.90% (73.5-95.8)	82.00% (68.6-91.4)

BLI, blue light imaging; CI, confidence interval; LCI, linked color imaging; NBI, narrow-band imaging; NPV, negative predictive value; PPV, positive predictive value; WLI, white light imaging

## Author Contribution Statement

The contributions of all authors must be described in the following manner: The authors confirm contribution to the paper as follows: study conception and design: X. Author, Y. Author; data collection: Y. Author; analysis and interpretation of results: X. Author, Y. Author. Z. Author; draft manuscript preparation: Y. Author. Z. Author. All authors reviewed the results and approved the final version of the manuscript.

## Conflicts of interest

The authors declare that they have no conflicts of interest.
